# 1-[(*Z*)-1-Bromo-2-(butyl­dichloro-λ^4^-tellan­yl)ethen­yl]cyclo­hex-1-ene

**DOI:** 10.1107/S1600536811023142

**Published:** 2011-06-18

**Authors:** Julio Zukerman-Schpector, Ignez Caracelli, Rafael Carlos Guadagnin, Hélio A. Stefani, Edward R. T. Tiekink

**Affiliations:** a Departmento de Química, Universidade Federal de São Carlos, CP 676, 13565-905 São Carlos, SP, Brazil; b BioMat-Departmento de Física, Universidade Federal de São Carlos, CP 676, 13565-905 São Carlos, SP, Brazil; c Departamento de Ciências Exatas e da Terra, Universidade Federal de São Paulo-Campus Diadema, Rua Prof. Artur Ridel 275, 09972-270 Diadema, SP, Brazil; d Departamento de Farmácia, Faculdade de Ciências Farmacêuticas, Universidade de São Paulo, São Paulo, SP, Brazil; e Department of Chemistry, University of Malaya, 50603 Kuala Lumpur, Malaysia

## Abstract

The Te^IV^ atom in the title compound, [Te(C_4_H_9_)(C_8_H_10_Br)Cl_2_] or C_12_H_19_BrCl_2_Te, is in a distorted ψ-trigonal–bipyramidal geometry, with the lone pair of electrons projected to occupy a position in the equatorial plane, and with the Cl atoms being mutually *trans* [172.48 (4)°]. Close intra­molecular [Te⋯Br = 3.3444 (18) Å] and inter­molecular [Te⋯Cl = 3.675 (3) Å] inter­actions are observed. The latter lead to centrosymmetric dimers which assemble into layers in the *bc* plane. The primary connections between layers are of the type C—H⋯Cl.

## Related literature

For background to the synthesis, see: Guadagnin *et al.* (2008 ▶). For related X-ray structures, see: Zukerman-Schpector *et al.* (1998 ▶, 2008 ▶). For coordination polyhedra around Te^IV^ atoms, see: Zukerman-Schpector & Haiduc (2002 ▶); Tiekink & Zukerman-Schpector (2010 ▶). For ring conformational analysis, see: Cremer & Pople (1975 ▶).
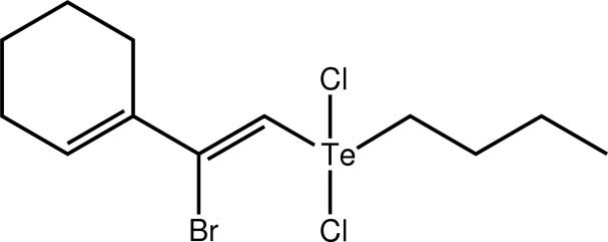


## Experimental

### 

#### Crystal data

C_12_H_19_BrCl_2_Te
*M* *_r_* = 441.67
Triclinic, 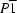

*a* = 6.311 (3) Å
*b* = 10.243 (6) Å
*c* = 12.334 (9) Å
α = 103.34 (2)°
β = 91.53 (2)°
γ = 91.411 (14)°
*V* = 775.1 (8) Å^3^
*Z* = 2
Mo *K*α radiation
μ = 4.82 mm^−1^
*T* = 98 K
0.22 × 0.20 × 0.15 mm

#### Data collection

Rigaku Saturn724 diffractometer
Absorption correction: multi-scan (*ABSCOR*; Higashi, 1995 ▶) *T*_min_ = 0.360, *T*_max_ = 0.486
7151 measured reflections
3012 independent reflections
2898 reflections with *I* > 2σ(*I*)
*R*_int_ = 0.033

#### Refinement

*R*[*F* ^2^ > 2σ(*F* ^2^)] = 0.032
*wR*(*F* ^2^) = 0.086
*S* = 1.12
3012 reflections
146 parameters
H-atom parameters constrained
Δρ_max_ = 0.89 e Å^−3^
Δρ_min_ = −0.59 e Å^−3^

### 

Data collection: *CrystalClear* (Molecular Structure Corporation & Rigaku, 2005 ▶); cell refinement: *CrystalClear*; data reduction: *CrystalClear*; program(s) used to solve structure: *SIR97* (Altomare *et al.*, 1999 ▶); program(s) used to refine structure: *SHELXL97* (Sheldrick, 2008 ▶); molecular graphics: *ORTEP-3* (Farrugia, 1997 ▶) and *DIAMOND* (Brandenburg, 2006 ▶); software used to prepare material for publication: *MarvinSketch* (Chemaxon, 2010 ▶) and *publCIF* (Westrip, 2010 ▶).

## Supplementary Material

Crystal structure: contains datablock(s) I, global. DOI: 10.1107/S1600536811023142/hg5055sup1.cif

Structure factors: contains datablock(s) I. DOI: 10.1107/S1600536811023142/hg5055Isup2.hkl

Supplementary material file. DOI: 10.1107/S1600536811023142/hg5055Isup3.cml

Additional supplementary materials:  crystallographic information; 3D view; checkCIF report

## Figures and Tables

**Table:** 

Te—Cl1	2.5381 (15)
Te—Cl2	2.4859 (15)
Te—C1	2.092 (4)
Te—C3	2.143 (4)

**Table:** 

Cl1—Te—Cl2	172.48 (4)

**Table 2:** Hydrogen-bond geometry (Å, °)

*D*—H⋯*A*	*D*—H	H⋯*A*	*D*⋯*A*	*D*—H⋯*A*
C3—H3a⋯Cl2^i^	0.97	2.80	3.576 (5)	138

Symmetry code: (i) 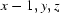.

**Fig. 1.**
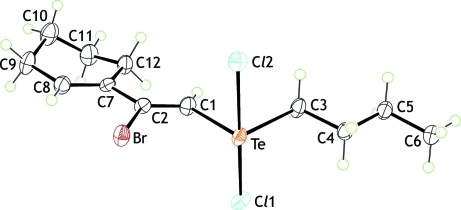
Molecular structure of (I) showing atom-labelling scheme and displacement ellipsoids at the 50% probability level.

**Fig. 2.**
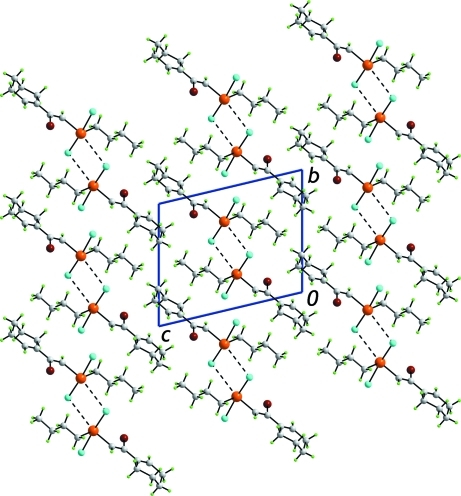
A view of the assembly of centrosymmetric molecules in the bc plane in (I). The intermolecular Te···Cl interactions are indicated by dashed lines.

**Fig. 3.**
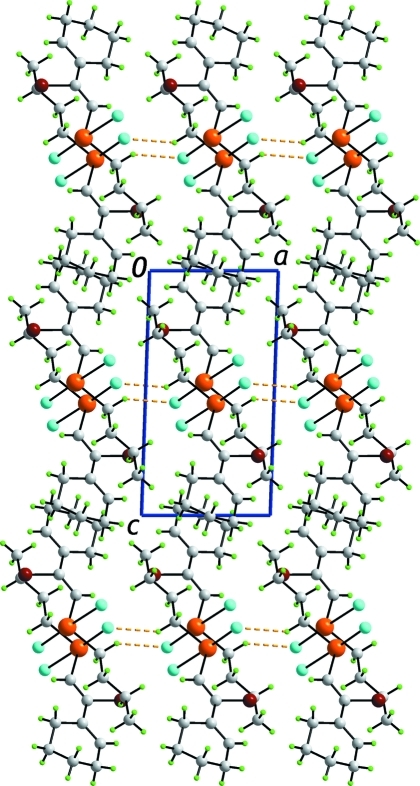
Unit-cell contents in (I) viewed in projection down the b axis showing C—H···Cl interactions (orange dashed lines) occurring between the layers shown in Fig. 2.

## supplementary crystallographic information

### Comment

The title compound, (I), was synthesized using a palladium-catalyzed cross-coupling reaction of a potassium aryltrifluoroborate salt with various (*Z*)-2-chloro vinylic tellurides (Guadagnin *et al.*, 2008). Complementing these studies are crystallographic studies (Zukerman-Schpector *et al.* 1998; Zukerman-Schpector *et al.*, 2008) focused upon determining coordination polyhedra and supramolecular aggregation patterns (Zukerman-Schpector & Haiduc, 2002; Tiekink & Zukerman-Schpector, 2010) which lead to the crystallographic characterization of (I).

The immediate coordination geometry about the Te^IV^ atom in (I) is defined by two Cl atoms and two C atoms which, along with a stereochemically active lone pair of electrons, define a ψ-trigonal bi-pyramidal geometry, Table 1. In this description the lone pair is assumed to occupy a position in the equatorial plane, and the Cl atoms to be mutually *trans*. Additional Te···*X* interactions are evident and contribute to the distortion of the coordination geometry. Thus, an intramolecular Te···Br interaction [3.3444 (18) Å] is noted. In addition, there is an intermolecular Te···Cl contact [Te···Cl1^i^ = 3.675 (3) Å, symmetry operation *i*: 1 - *x*, 1 - *y*, 1 - *z*]. The latter interaction explains the elongation of the Te—Cl1 bond compared to the Te—Cl2 bond, Table 1. Within the substituted ligand, the configuration about the C1═C2 bond [1.327 (6) Å] is *Z*. The cyclohexene ring adopts a half-chair conformation with puckering parameters: q_2_ = 0.364 (5) Å and q_3_ = -0.278 (5) Å, and amplitudes: Q = 0.458 (6) Å, θ = 127.4 (6) ° and φ_2_ = 30.2 (8) ° (Cremer & Pople, 1975).

The most prominent feature of the crystal packing are the aforementioned Te···Cl1 interactions. These lead to centrosymmetric dimers that assemble into chains along the *b* axis. These partially inter-digitate along the *c* axis. The layers thus formed in the *bc* plane, Fig. 2, are connected *via* C—H···Cl interactions, Table 2, along the *a* axis, Fig. 3.

### Experimental

The starting (*Z*)-(2-bromo-2-cyclohexenylvinyl)(butyl)tellane was prepared as described in previous work (Guadagnin *et al.*, 2008) and a solution of it (1 mmol, 0.370 g) in hexane (5 ml) at 273 K was poured into a two-necked round-bottomed flask under a nitrogen atmosphere and then SO_2_Cl_2_ (1 mmol, 1.37 g) added drop wise. A white solid formed immediately. The mixture was warmed to room temperature. The resulting solid was filtered and dried. Crystals of (I) were obatined by slow evaporation from its CHCl_3_ solution held at room temperature.

### Refinement

C-bound H-atoms were placed in calculated positions (C—H 0.93–0.97 Å) and were included in the refinement in the riding model approximation with *U*_iso_(H) set to 1.2–1.5*U*_eq_(C).

### Crystal data

**Table:**

C_12_H_19_BrCl_2_Te	*Z* = 2
*M**_r_* = 441.67	*F*(000) = 424
Triclinic, *P*1	*D*_x_ = 1.893 Mg m^−^^3^
Hall symbol: -P 1	Mo *K*α radiation, λ = 0.71073 Å
*a* = 6.311 (3) Å	Cell parameters from 2823 reflections
*b* = 10.243 (6) Å	θ = 2.3–30.3°
*c* = 12.334 (9) Å	µ = 4.82 mm^−^^1^
α = 103.34 (2)°	*T* = 98 K
β = 91.53 (2)°	Block, colourless
γ = 91.411 (14)°	0.22 × 0.20 × 0.15 mm
*V* = 775.1 (8) Å^3^	

### Data collection

**Table:**

Rigaku Saturn724 diffractometer	3012 independent reflections
Radiation source: fine-focus sealed tube	2898 reflections with *I* > 2σ(*I*)
graphite	*R*_int_ = 0.033
Detector resolution: 28.5714 pixels mm^-1^	θ_max_ = 26.0°, θ_min_ = 3.0°
dtprofit.ref scans	*h* = −7→7
Absorption correction: multi-scan (*ABSCOR*; Higashi, 1995)	*k* = −12→12
*T*_min_ = 0.360, *T*_max_ = 0.486	*l* = −15→14
7151 measured reflections	

### Refinement

**Table:**

Refinement on *F*^2^	Primary atom site location: structure-invariant direct methods
Least-squares matrix: full	Secondary atom site location: difference Fourier map
*R*[*F*^2^ > 2σ(*F*^2^)] = 0.032	Hydrogen site location: inferred from neighbouring sites
*wR*(*F*^2^) = 0.086	H-atom parameters constrained
*S* = 1.12	*w* = 1/[σ^2^(*F*_o_^2^) + (0.0328*P*)^2^ + 2.7479*P*] where *P* = (*F*_o_^2^ + 2*F*_c_^2^)/3
3012 reflections	(Δ/σ)_max_ < 0.001
146 parameters	Δρ_max_ = 0.89 e Å^−^^3^
0 restraints	Δρ_min_ = −0.59 e Å^−^^3^

### Special details

**Table:**

Geometry. All s.u.'s (except the s.u. in the dihedral angle between two l.s. planes) are estimated using the full covariance matrix. The cell s.u.'s are taken into account individually in the estimation of s.u.'s in distances, angles and torsion angles; correlations between s.u.'s in cell parameters are only used when they are defined by crystal symmetry. An approximate (isotropic) treatment of cell s.u.'s is used for estimating s.u.'s involving l.s. planes.
Refinement. Refinement of *F*^2^ against ALL reflections. The weighted *R*-factor *wR* and goodness of fit *S* are based on *F*^2^, conventional *R*-factors *R* are based on *F*, with *F* set to zero for negative *F*^2^. The threshold expression of *F*^2^ > 2σ(*F*^2^) is used only for calculating *R*-factors(gt) *etc*. and is not relevant to the choice of reflections for refinement. *R*-factors based on *F*^2^ are statistically about twice as large as those based on *F*, and *R*- factors based on ALL data will be even larger.

### Fractional atomic coordinates and isotropic or equivalent isotropic displacement parameters (Å^2^)

**Table:**

	*x*	*y*	*z*	*U*_iso_*/*U*_eq_	
Te	0.54785 (4)	0.72646 (3)	0.54317 (2)	0.02297 (11)	
Cl1	0.28881 (18)	0.59324 (11)	0.63286 (10)	0.0303 (2)	
Cl2	0.76717 (17)	0.88070 (11)	0.46001 (9)	0.0270 (2)	
Br	0.88870 (7)	0.84752 (4)	0.75713 (4)	0.02781 (13)	
C1	0.4888 (7)	0.8911 (4)	0.6734 (4)	0.0243 (9)	
H1	0.3606	0.9336	0.6721	0.029*	
C2	0.6258 (6)	0.9377 (4)	0.7578 (4)	0.0210 (8)	
C3	0.2891 (7)	0.7512 (5)	0.4343 (4)	0.0272 (10)	
H3A	0.1601	0.7647	0.4760	0.033*	
H3B	0.3167	0.8303	0.4056	0.033*	
C4	0.2579 (7)	0.6278 (4)	0.3363 (4)	0.0254 (9)	
H4A	0.3884	0.6127	0.2960	0.031*	
H4B	0.2263	0.5491	0.3649	0.031*	
C5	0.0798 (7)	0.6463 (5)	0.2574 (4)	0.0275 (9)	
H5A	0.1144	0.7224	0.2260	0.033*	
H5B	−0.0492	0.6655	0.2983	0.033*	
C6	0.0426 (8)	0.5215 (5)	0.1634 (4)	0.0327 (11)	
H6A	0.1697	0.5029	0.1223	0.049*	
H6B	−0.0705	0.5363	0.1145	0.049*	
H6C	0.0051	0.4465	0.1942	0.049*	
C7	0.5937 (7)	1.0547 (4)	0.8505 (4)	0.0218 (9)	
C8	0.7386 (8)	1.0984 (5)	0.9333 (4)	0.0278 (10)	
H8	0.8617	1.0502	0.9330	0.033*	
C9	0.7148 (8)	1.2201 (5)	1.0265 (4)	0.0339 (11)	
H9A	0.8450	1.2744	1.0361	0.041*	
H9B	0.6925	1.1917	1.0952	0.041*	
C10	0.5313 (8)	1.3048 (5)	1.0050 (5)	0.0390 (12)	
H10A	0.4959	1.3655	1.0744	0.047*	
H10B	0.5742	1.3585	0.9536	0.047*	
C11	0.3404 (8)	1.2207 (5)	0.9576 (4)	0.0358 (11)	
H11A	0.2274	1.2786	0.9452	0.043*	
H11B	0.2927	1.1710	1.0110	0.043*	
C12	0.3841 (7)	1.1224 (5)	0.8482 (4)	0.0280 (10)	
H12A	0.2710	1.0542	0.8316	0.034*	
H12B	0.3833	1.1700	0.7888	0.034*	

### Atomic displacement parameters (Å^2^)

**Table:**

	*U*^11^	*U*^22^	*U*^33^	*U*^12^	*U*^13^	*U*^23^
Te	0.02153 (17)	0.01990 (16)	0.02365 (17)	0.00270 (11)	−0.00238 (11)	−0.00268 (11)
Cl1	0.0311 (6)	0.0213 (5)	0.0360 (6)	−0.0017 (4)	0.0052 (5)	0.0010 (4)
Cl2	0.0254 (5)	0.0286 (5)	0.0260 (5)	0.0036 (4)	0.0036 (4)	0.0034 (4)
Br	0.0224 (2)	0.0283 (2)	0.0286 (2)	0.00692 (18)	−0.00469 (18)	−0.00199 (18)
C1	0.023 (2)	0.023 (2)	0.025 (2)	0.0068 (17)	0.0015 (17)	−0.0014 (17)
C2	0.0174 (19)	0.0191 (19)	0.026 (2)	0.0021 (16)	0.0012 (16)	0.0042 (17)
C3	0.023 (2)	0.024 (2)	0.030 (2)	0.0023 (17)	−0.0078 (18)	−0.0023 (19)
C4	0.032 (2)	0.021 (2)	0.021 (2)	0.0082 (18)	−0.0013 (18)	0.0000 (17)
C5	0.031 (2)	0.024 (2)	0.025 (2)	0.0000 (18)	−0.0044 (18)	0.0004 (18)
C6	0.042 (3)	0.028 (2)	0.025 (2)	0.000 (2)	−0.004 (2)	0.0016 (19)
C7	0.026 (2)	0.0157 (19)	0.024 (2)	−0.0026 (16)	0.0001 (17)	0.0042 (16)
C8	0.028 (2)	0.025 (2)	0.028 (2)	0.0017 (18)	−0.0027 (19)	0.0029 (19)
C9	0.031 (3)	0.033 (3)	0.030 (2)	0.000 (2)	−0.003 (2)	−0.009 (2)
C10	0.040 (3)	0.032 (3)	0.037 (3)	0.005 (2)	0.000 (2)	−0.008 (2)
C11	0.034 (3)	0.034 (3)	0.034 (3)	0.010 (2)	0.000 (2)	−0.001 (2)
C12	0.026 (2)	0.027 (2)	0.027 (2)	0.0040 (18)	−0.0047 (18)	0.0000 (19)

### Geometric parameters (Å, °)

**Table:**

Te—Cl1	2.5381 (15)	C6—H6B	0.9600
Te—Cl2	2.4859 (15)	C6—H6C	0.9600
Te—C1	2.092 (4)	C7—C8	1.341 (6)
Te—C3	2.143 (4)	C7—C12	1.511 (6)
Br—C2	1.918 (4)	C8—C9	1.502 (6)
C1—C2	1.327 (6)	C8—H8	0.9300
C1—H1	0.9300	C9—C10	1.518 (7)
C2—C7	1.475 (6)	C9—H9A	0.9700
C3—C4	1.539 (6)	C9—H9B	0.9700
C3—H3A	0.9700	C10—C11	1.488 (7)
C3—H3B	0.9700	C10—H10A	0.9700
C4—C5	1.511 (6)	C10—H10B	0.9700
C4—H4A	0.9700	C11—C12	1.522 (7)
C4—H4B	0.9700	C11—H11A	0.9700
C5—C6	1.524 (6)	C11—H11B	0.9700
C5—H5A	0.9700	C12—H12A	0.9700
C5—H5B	0.9700	C12—H12B	0.9700
C6—H6A	0.9600		
			
C1—Te—C3	97.25 (17)	H6A—C6—H6C	109.5
C1—Te—Cl2	87.77 (14)	H6B—C6—H6C	109.5
C3—Te—Cl2	88.63 (14)	C8—C7—C2	122.8 (4)
C1—Te—Cl1	86.80 (14)	C8—C7—C12	121.3 (4)
Cl1—Te—Cl2	172.48 (4)	C2—C7—C12	115.9 (4)
C2—C1—Te	123.1 (3)	C7—C8—C9	124.2 (4)
C2—C1—H1	118.5	C7—C8—H8	117.9
Te—C1—H1	118.5	C9—C8—H8	117.9
C1—C2—C7	125.3 (4)	C8—C9—C10	112.4 (4)
C1—C2—Br	117.1 (3)	C8—C9—H9A	109.1
C7—C2—Br	117.6 (3)	C10—C9—H9A	109.1
C4—C3—Te	111.2 (3)	C8—C9—H9B	109.1
C4—C3—H3A	109.4	C10—C9—H9B	109.1
Te—C3—H3A	109.4	H9A—C9—H9B	107.9
C4—C3—H3B	109.4	C11—C10—C9	111.9 (4)
Te—C3—H3B	109.4	C11—C10—H10A	109.2
H3A—C3—H3B	108.0	C9—C10—H10A	109.2
C5—C4—C3	111.5 (4)	C11—C10—H10B	109.2
C5—C4—H4A	109.3	C9—C10—H10B	109.2
C3—C4—H4A	109.3	H10A—C10—H10B	107.9
C5—C4—H4B	109.3	C10—C11—C12	112.2 (4)
C3—C4—H4B	109.3	C10—C11—H11A	109.2
H4A—C4—H4B	108.0	C12—C11—H11A	109.2
C4—C5—C6	111.3 (4)	C10—C11—H11B	109.2
C4—C5—H5A	109.4	C12—C11—H11B	109.2
C6—C5—H5A	109.4	H11A—C11—H11B	107.9
C4—C5—H5B	109.4	C7—C12—C11	113.1 (4)
C6—C5—H5B	109.4	C7—C12—H12A	109.0
H5A—C5—H5B	108.0	C11—C12—H12A	109.0
C5—C6—H6A	109.5	C7—C12—H12B	109.0
C5—C6—H6B	109.5	C11—C12—H12B	109.0
H6A—C6—H6B	109.5	H12A—C12—H12B	107.8
C5—C6—H6C	109.5		
			
C3—Te—C1—C2	−168.3 (4)	Br—C2—C7—C8	−0.7 (6)
Cl2—Te—C1—C2	−80.0 (4)	C1—C2—C7—C12	1.7 (6)
Cl1—Te—C1—C2	105.2 (4)	Br—C2—C7—C12	−179.7 (3)
Te—C1—C2—C7	178.8 (3)	C2—C7—C8—C9	177.6 (4)
Te—C1—C2—Br	0.2 (5)	C12—C7—C8—C9	−3.5 (7)
C1—Te—C3—C4	−168.9 (3)	C7—C8—C9—C10	−11.7 (7)
Cl2—Te—C3—C4	103.5 (3)	C8—C9—C10—C11	42.3 (6)
Cl1—Te—C3—C4	−82.6 (3)	C9—C10—C11—C12	−58.9 (6)
Te—C3—C4—C5	−178.3 (3)	C8—C7—C12—C11	−12.0 (6)
C3—C4—C5—C6	−177.4 (4)	C2—C7—C12—C11	167.0 (4)
C1—C2—C7—C8	−179.3 (5)	C10—C11—C12—C7	42.8 (6)

### Hydrogen-bond geometry (Å, °)

**Table:**

*D*—H···*A*	*D*—H	H···*A*	*D*···*A*	*D*—H···*A*
C3—H3a···Cl2^i^	0.97	2.80	3.576 (5)	138

Symmetry codes: (i) *x*−1, *y*, *z*.
